# Canadian Women's Experience of Postnatal Care: A Mixed Method Study

**DOI:** 10.1177/08445621211052141

**Published:** 2021-10-27

**Authors:** Justine Dol, Brianna Hughes, Gail Tomblin Murphy, Megan Aston, Douglas McMillan, Marsha Campbell-Yeo

**Affiliations:** 1 Faculty of Health, 3688 Dalhousie University, Halifax, NS, Canada; 2 School of Nursing, Faculty of Health, 70338 Dalhousie University, Halifax, NS, Canada; 3 432234 Nova Scotia Health Authority, Halifax, NS, Canada; 4 Division of Neonatal Perinatal Medicine, Department of Pediatrics, Faculty of Medicine, 70338 Dalhousie University and IWK Health Centre, Halifax, NS, Canada

**Keywords:** postpartum period, postnatal care, mothers, infant, newborn

## Abstract

**Background:**

The postnatal period remains unstandardized in terms of care and postnatal visits with a dearth of information on the experience from Canadian women.

**Purpose:**

To explore (1) with whom and how often women receive postnatal follow-up visits and (2) the postnatal care experiences of Canadian mothers.

**Methods:**

Using a cross-sectional design, women who had given birth within the past 6 months were recruited to complete an online survey. Frequencies were computed for quantitative outcomes and thematic analysis was used for qualitative responses.

**Results:**

A total of 561 mothers completed the survey. Women saw on average 1.9 different postnatal healthcare providers, primarily family doctors (72.4%). 3.2% had no postnatal visits and 37.6% had 4  or more within 6 weeks. 76.1% women were satisfied with their postnatal care. Women's satisfactory care in the postnatal period was associated with in-person and at home follow-ups, receiving support, and receiving timely, appropriate care for self and newborn. Unsatisfactory care was associated with challenges accessing care, experiencing gaps in follow-up visits, and having unsatisfactory assessment for their own recovery.

**Conclusion:**

There is considerable variation in the timing and frequency of postnatal visits. While many women are experiencing satisfactory care, women are still reporting dissatisfaction and are facing challenges.

## Background and Purpose

Despite the significant challenges and changes that occur in the period after birth for a woman and her newborn, standardized healthcare in the postnatal period is limited (Verbiest et al., 2016). The lack of formal, standardized support during the postnatal period for women and their newborn is a shift from the intense, routine monitoring that occurs near the end of pregnancy where women have appointments with healthcare providers often on a weekly basis (Tully et al., 2017). The quality of care a woman receives after birth varies depending on the type of care provider involved in her care, where the woman lives, whether they are considered high risk, or have identified health concerns for themselves or their newborn (Reproductive Care Program of Nova Scotia, 2002; Verbiest et al., 2016). There is evidence that women considered healthy or low risk also face challenges in the postpartum period (Shorey et al., 2018), in addition well-established concerns around high risk (Dennis et al., 2016; Norhayati et al., 2015) or vulnerable populations (Daoud et al., 2019; Falah-Hassani et al., 2015; Haque et al., 2017), yet little is known about their postpartum experiences.

The World Health Organization (WHO) recommends that postnatal care for newborns and women occur within the first 24 hours (h) after birth, followed by a contact with a healthcare provider between 48 and 72 h, 7–14 days, and 6 weeks post birth (World Health Organization, 2013). In Canada, recommendations by the Canadian Paediatric Society are for the newborn to be reassessed between 48 and 72 h (Lemyre et al., 2018) and at 1 week (Canadian Paediatric Society, 2020). The Society of Obstetricians and Gynecologists of Canada (SOGC) (2020) recommends that women and their newborns be followed-up at 1 week and 4–6 weeks post birth. However, there is a lack of consistent methods of implementation of postnatal care, education or support within Canada, with recommendations for postnatal care varying by province and provider, despite detailed public health protocols that provide guidance in terms of supports, contacts, and home visits (Public Health Agency of Canada, 2020; Reproductive Care Program of Nova Scotia, 2002). While postpartum people should have standardized access to doctors, nurses, and midwives who can provide information about new experiences and caring for their infant, there is a paucity of information on the number and timing of postnatal visits, optimal provider type, and whether the current models meet women's needs (Tully et al., 2017; World Health Organization, 2010).

Canada has a universal health care system in which access to perinatal care is free to all Canadian citizens and permanent residents who apply for public health insurance (Government of Canada, 2020). Despite the universal healthcare system, healthcare is managed provincially with each province having their own insurance plan that regulates and oversees healthcare (Government of Canada, 2020). In Nove Scotia (NS), recommendations are for a postnatal care contact within 1–2 weeks after birth, followed by another follow-up at 6 weeks (Reproductive Care Program of Nova Scotia, 2002). However, both New Brunswick (NB) and Prince Edward Island (PEI) do not have any standardized recommendations for postnatal visits, with timing and frequency of follow-ups varying depending on the care provider (personal communication, NB and PEI Public Health representatives). A focus on the Maritime provinces was chosen to add to the existing literature on postpartum care and outcomes across Canada (Dennis et al., 2016; Kingston et al., 2018; Sword et al., 2009).

There is significant paucity of information on the overall postpartum experience of Canadian women. The most recent Canadian evaluation of postpartum care was part of the 2006 Maternity Experiences Survey with 6,421 women across Canada (Chalmers et al., 2008; Public Health Agency of Canada, 2009). They reported that 93.9% of women were contacted by a healthcare provider on average 6.8 days after birth, but this varied by province (Chalmers et al., 2008; Public Health Agency of Canada, 2009). They also found that two-thirds of women were very satisfied with the care that they received, and three-quarters were very satisfied with the care their newborn received (Chalmers et al., 2008; Public Health Agency of Canada, 2009). However, they did not report on what resulted in a satisfactory versus unsatisfactory care experience nor did they provide detailed information on which healthcare providers were involved in postnatal care.

## Problem Statement

Currently, there is a lack of national consensus recommendations related to the provision of postnatal follow-up as well as paucity of evidence of women's perceived degree of satisfaction regarding postpartum care.

## Purpose Statement

To address the gap in our understanding of the postpartum experience of Canadian women, both in terms of access and use of healthcare providers as well as their perceptions on current provision of postpartum care, this study aims to answer the following questions: (1) which healthcare providers are providing care to women in the postpartum period, (2) how frequently do women and infants receive postnatal follow-up visits, and (3) how are women experiencing satisfaction (or lack therefore) related to their postnatal care? Therefore, the first objective of the current study is to explore with whom and how often women receive postnatal follow-up visits. The second objective is to explore reasons for satisfaction and unsatisfaction with current postnatal care for women living in the Maritime provinces.

## Methods and Procedures

### Study Design

This study used a cross-sectional design with a mixed-method survey approach. The survey was developed using both questionnaires on healthcare provider usage as well as open-ended questions. This study is part of a larger study exploring the postpartum experience of women living in the Maritime provinces.

### Setting and Sample

People who had given birth within the past 6 months and lived in either NS, NB, or PEI were recruited. Women must have given birth between April 1, 2019 and January 1, 2020. These three provinces cover 133,850 km^2^ and represent 5.1% of Canada's total population (Statistics Canada, 2019). The annual number of births in these provinces was 15,684 in 2018, representing 4.2% of Canadian births (Statistics Canada, n.d.).

To be eligible, women must have met the following criteria: (1) have a newborn 6 months of age or less; (2) be over 18 years of age; (3) be able to speak, write, and read English; and (4) currently live in either NS, NB, or PEI. Mothers were excluded if they lived outside these provinces or had their most recent baby over 6 months prior to survey completion. There was no limit on the number of survey responses but a targeted sample size of 375 mothers was estimated to be sufficient with a margin of error of 5% and level of confidence of 95% using a population estimate of 15,684 women in these provinces giving birth in a 12-month period (Sample Size Calculator, 2014).

### Protection of Human Subjects

Institutional ethical approval was obtained from IWK Health research ethics board prior to data collection. Participants were informed during the consent statement that they could stop the survey at any time by exiting the survey. Participants who did not complete the full survey were not included in the analysis. Data was deidentified prior to analysis to ensure confidentiality and secure electronic storage. After completing the survey, participants were provided a list of national and local resources for postpartum support if needed.

### Data Collection Procedures

Women were asked to participate in an online survey via social media, including boosted posts (paid advertisement) on Facebook, as well as media releases, posters, and word of mouth. The survey was open for a 3-month period (October 1, 2019 to January 1, 2020) and was hosted on a secure university survey platform. Prior to starting the survey, participants completed an online consent form and eligibility questionnaire to ensure they met all inclusion criteria outlined above. Once participants entered the survey, demographics were collected and participants completed the survey. All questions relevant to the current study are available in Table 1. The survey took approximately 30 min to complete, and participants could opt into a draw for one of three $100 CAN electronic gift cards once they completed the full survey.

**Table 1.** Questions Asked in the Survey.

Question	Available response options
During your pregnancy, which type of caregiver was most directly involved in providing you care?	An obstetrician-gynecological doctor
	A family doctor
	A midwife
	A nurse practitioner
	A nurse who was not a midwife
	Other
	Not sure
	Prefer not to answer
During the postnatal period, which type of healthcare provider did you receive care from (check all that apply)?	An obstetrician-gynecological doctor
	A family doctor
	A midwife
	A nurse practitioner
	A nurse who was not a midwife
	Other
	Not sure
	Prefer not to answer
After your baby was born, how many times did you see a healthcare provider within the first 6 weeks?	0
	1
	2
	3
	4 +
	Not applicable
	Prefer not to answer
When did you visit the healthcare provider? (select all that apply)	Day 1–2
	Day 3–7
	Day 8–14
	Day 15–21
	Day 22–28
	Day 29–36
	Day 37–42
	Day 43–49
	Don’t know/remember
	Not applicable
	Prefer not to answer
Were you satisfied with the care you received postnatally?	Yes
	No
	Don’t know/remember
	Prefer not to answer
Please describe your experiences with postnatal care interactions with healthcare providers.	Open ended

Of note, the terms “postnatal” and “postpartum” are often used interchangeably in the literature, with the former referring to the newborn and the latter referring to the women, with the term postnatal typically used pertaining to the dyad (World Health Organization, 2010). For the purposes of this study, we used the terms in the same way. Similarly, while the survey was open to all birthing people and while recognizing that not all birthing people identify as women, only people who identified as women participated, thus the terms “women” and “mother” are used throughout.

### Data Analysis Procedures

Quantitative data were analyzed using the statistical package for social sciences (SPSS 25). Total scores and frequencies were computed for all relevant outcomes and Chi square tests were used to explore differences between primiparous and multiparous women on quantitative outcomes. Qualitative data was analyzed using Nvivo 12 using thematic analysis informed by a qualitative descriptive approach (Bradshaw et al., 2017; Braun & Clarke, 2006), led by the first author and verified by the second author. Codes were applied to responses and grouped within larger themes under the larger categories of satisfactory care and unsatisfactory care. Once codes were developed and defined, all quotes were reviewed by the first author to ensure they were applied consistently. The second author then reviewed all codes and themes for consistency and verification. Minor discrepancies were discussed, and codes revised through discussion until an agreement was reached for all coded responses.

## Results

Five-hundred and sixty-one (561) mothers completed all aspects of the survey, which was 73.7% of all respondents who self-identified as eligible and completed the consent form (*n* = 761). There was representation across the provinces, with 46.5% of mothers from NS, 30.5% from NB, and 23.0% from PEI.

Mothers were on average 30.7 years of age (standard deviation (SD)  =  4.6) with a range of 1–5 biological children (*M* = 1.6, SD  =  0.8). Over half of the women (56.5%) were primiparous. Additional demographic details about the sample are included in Table 2.

**Table 2.** Demographics of Participants.

Demographics	Participants (*n* = 561) *n* (%)
Infant age	
0–3 months	309 (55.1)
4–6 months	252 (44.9)
Parity	
Primiparous	317 (56.5)
Multiparous	244 (43.5)
Marital status	
Single	12 (2.1)
In a relationship	67 (12.0)
Married or common law	479 (85.4)
Missing	3 (0.5)
Household income (CAN)	
Less than $44,000	100 (17.8)
$45,000–$139,000	322 (57.4)
Over $140,000–$199,000	108 (19.3)
Missing	31 (5.5)
Baby birth weight	
Under 2,500 g	30 (5.4)
2,500–4,000 g	360 (64.2)
Over 4,000 g	96 (17.1)
Missing	75 (13.3)
Birth method	
Vaginal birth	407 (72.5)
Unplanned cesarean	102 (18.2)
Planned cesarean	52 (9.3)

### Survey Results on Postnatal Care Providers and Frequency

In terms of the healthcare provider involved in their care, most women were followed during their pregnancy by an obstetrician (56.9%) or a family doctor (26.6%, Table 3). In the postpartum period, women were primarily followed by a family doctor (72.4%) and less often by an obstetrician (55.8%). For these outcomes, respondents could select more than one healthcare provider who was involved in their care. There were no differences between primiparous and multiparous women on which healthcare providers were involved in perinatal care. Women saw on average 1.9 healthcare providers during the postpartum period (SD  =  0.90, range 0–4).

**Table 3.** All Healthcare Providers Providing Perinatal Care.

Healthcare provider	Pregnancy *n* (%)	Postnatal^a^ *n* (%)
An obstetrician	319 (56.9)	313 (55.8)
A family doctor	149 (26.6)	407 (72.5)
A midwife	18 (3.2)	17 (3.0)
A nurse practitioner	51 (9.1)	138 (24.6)
A nurse who was not a midwife	11 (2.0)	152 (27.1)
Other	13 (2.2)	28 (5.0)

^a^ Does not equal 100% due to the ability to select more than one option.

**Figure 1.**
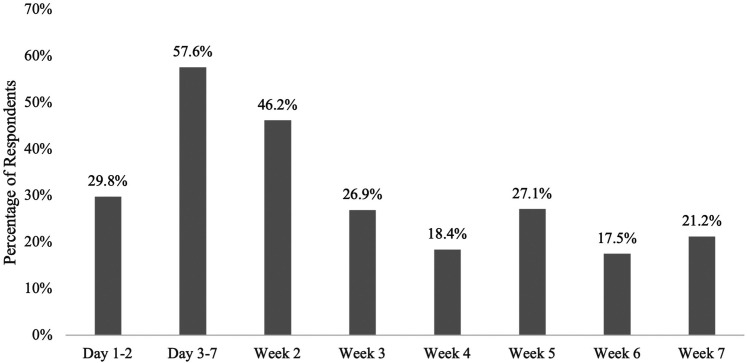
Percentage of women who had a postnatal visit with a healthcare provider during postpartum week.

The number of visits that women had with any healthcare provider within the first 6 weeks varied: 3.2% had no postnatal visits, 15.2% had one visit, 21.6% had two or three visits each, and 37.6% had four or more. Of the 538 women who had a visit (excluding respondents who said not applicable or prefer not to answer), there was also variation when these women visited a healthcare provider. Over half of the women had a postpartum contact between day 3–7 (57.6%) while 46.2% reported having an appointment during day 8–14 (Figure 1). Less than a third of women reporting having a postpartum visit at each of the following weekly time-points, with the lowest follow-up between day 37–42 (17.5%). There were no significant differences between primiparous and multiparous women in terms of the number of visits, however, primiparous women were more significantly likely to have a postnatal appointment during week 3 (*X*^2^  =  5.924, *p*  =  .015), week 4 (*X*^2^  =  5.642, *p*  =  .018), and week 6 (*X*^2^  =  5.678, *p*  =  .017).

**Figure 2.**
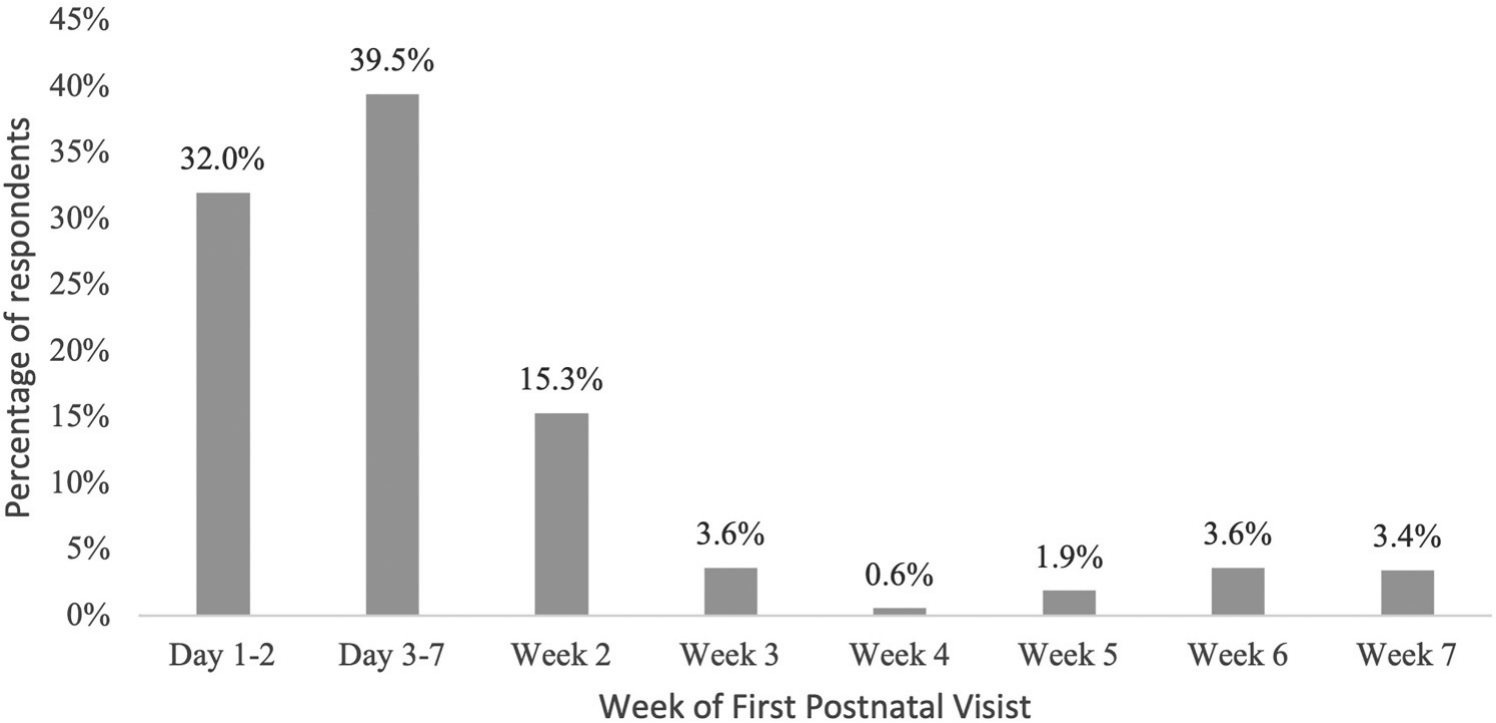
Timing of when women had their first postnatal visit with a healthcare provider.

Of those who had at least one follow-up contact, their initial postnatal visit with a health care provider was within the first week—32.0% (*n* = 167) in the first 2 days and 39.5% (*n* = 206) between day 3 and 7 (Figure 2). Seven percent had their first follow-up visit in either week 6 or week 7.

### Qualitative Themes Related to Postnatal Care Experiences

When asked if women were satisfied with the care they received during their postnatal follow-ups, 76.1% of women responded that they were satisfied. Twenty percent were unsatisfied and 3.9% preferred not to answer. Almost two-thirds (*n* = 357, 63.6%) of women provided some details about her postnatal experience when provided an opportunity to respond to an open-ended question. From these responses, six themes were identified—three themes related to satisfactory care and three themes related to unsatisfactory care. For satisfactory care, women (1) valued in-person follow-up from public health nurses and midwives; (2) received support from their healthcare providers; and (3) perceived receiving timely, appropriate care. For unsatisfactory care, women (1) had challenges accessing postnatal care; (2) experienced gaps in postnatal follow-ups; and (3) had unsatisfactory postpartum checks for themselves on their physical recovery.

### Satisfactory Care

The first theme that was identified by women who experienced satisfactory care was that the in-person follow-up provided by public health nurses and midwives was valued. Women acknowledged that having the public health nurses come to their homes was an invaluable part of their postnatal experience: “Thank goodness for the public health nurses who would visit and help us within the first few weeks of weight gain trouble and anxiety.” Women appreciated their visits over the first 6 weeks and the help they provided with breastfeeding and weighing their baby. Women who were under the care of the midwives, although a small percentage of the sample, also acknowledged the benefit of having them check in on them as well as the ability to contact them directly with any questions they have. One woman said:

Midwifery care provides excellent postpartum care. I was reassured to know that I could page them at any time with concerns. I think the immediate postpartum period would have been a lot more difficult without midwifery care. This support is needed in such a crucial moment in the mother-baby dyad.

The second theme related to satisfactory care was when women received support from their healthcare providers. Women felt that healthcare providers were generally helpful: “my physician and nurse [were] beyond kind and helpful” and “very thorough, took time to update me on new best-practice procedures since my last baby.” In particular, women commented that healthcare providers were helpful in answering their breastfeeding questions and providing support for breastfeeding issues: “the nurses were wonderful at helping me to breastfeed and building my confidence feeding my baby.” Another woman said: “[I] found the public health nurse extremely helpful, she came to my house multiple times to help with breastfeeding and pumping. I saw a lactation consultant who I also found very helpful.” Women felt that their healthcare provider was able to answer any questions they had and provide resources and more information. One woman said her healthcare provider was “helpful in providing information and answering questions without judgement or attitude.” Women also felt supported and reassured by their healthcare provider, both in terms of anxiety and uncertainty around infant care but also physical changes they were experiencing. Women who felt satisfied felt the knowledge of their healthcare provider was sufficient and they were able to provide advice and care to themselves and their newborn.

The final theme for women who were satisfied with their care was that they received timely, appropriate follow-ups. Women commented on their ability to receive postnatal appointments during an appropriate time frame: “My family doctor was excellent, got me right into an appointment within five days after my daughter was born.” Another woman said: “Our family doctor was also fantastic, providing a check-up of the baby and myself at two weeks postnatal and four weeks postnatal.” Women also appreciated that their healthcare provider asked about their mental and physical health: “Extremely attentive and professional and again, asked appropriate questions regarding mental and physical health” and “always asked about both my physical and mental states and checked on baby as well. Felt very taken care of.” Women also mentioned that the 6-week appointment was often a time where contraception and family planning was discussed and offered.

### Unsatisfactory Care

While many women had positive experiences, women also had challenges accessing postnatal care, resulting in an unsatisfactory care experience. In particular, women commented on their difficulty in getting follow-up appointments, despite reaching out for support. Some examples of challenges in getting appointments for newborn care are as follows:

I did try to receive [hospital] breastfeeding support once we learned baby was not gaining weight sufficiently. I left three messages and called multiple times and was unable to reach anyone for support.

… due to long wait times to see my doctor, my newborn did not get in for the two week check that was suggested when I left the hospital. He was nearly six weeks old when he was first seen.

Women also experienced challenges getting their own personal follow-up appointments as well:

I did have to wait 12 weeks for my 6-week follow up with my obstetrician [OB] because of shortage of OBs at a near hospital.

[I] have been having issues with a lot of pain in my tailbone. Went to the ER one night because I could not get an appointment with a doctor about it.

Women also discussed the long wait times for appointments or delays in appointments once booked. One woman explained: “She has some feeding problems with spitting up and [I have] been waiting 2 months for a call from the pediatrician which I feel is a long time to wait when it comes to the feeding and health of a newborn baby.” Another woman said: “After having to wait over an hour in a room full of sick people with a newborn, I then had to wait another 45 minutes in a room waiting for the doctor.”

The second theme that emerged related to unsatisfied care during the postnatal period was that women experienced gaps in care during follow-ups: “[My follow-up was] brief, [I] would have preferred a checkup 2 weeks postpartum as well as 6 weeks. Rather than googling everything for 6 weeks.” Women felt that they wanted more information than was often provided, with some mothers also mentioning that healthcare providers offered conflicting information.

The public health nurse visited our home twice. Her information often differed from what the nurses said in the hospital. There was an inconsistency in the information provided.

Another challenge noted was that some women felt pressured by their healthcare provider to engage in certain behaviors without listening to their needs, particularly around breastfeeding.

The attitude and anxiety from medical professionals that I felt and received about supplementing with formula when I had supply issues and he was losing weight really caused significant mental distress for me.

Women reported disappointment in the care received, both in the hospital in the immediate postpartum period as well as in their follow-up care: “It was very vague, and I felt that she did not seem to care.” Not only were some women disappointed, some felt they received inadequate care, both for themselves and their infant:

Felt like my particular needs, concerns and problems were not really understood and [I] was just repeated the same advice over and over without any real help.

Someone came to take the baby's blood and it took a long time and the baby was screaming for the entire duration. We were not given the option to comfort the baby during this procedure. My husband and I found that very stressful.

Women felt rushed or ignored in their appointments, that their healthcare provider did not have time for their concerns, that their concerns were not valid, or they did not have time for any questions.

There should be a lot more hospital visits with your OB after you have a baby. I feel like when you’re pregnant, you get an appointment every month / week. Then after baby, you are kind of forgotten about and no one cares as much.

This was also in reference to their physical healing after birth—women felt that their healthcare provider did not provide a physical check on their healing, particularly for women who had caesarean sections, and were more concerned about the baby than the mother. A mother explained: “No one asked about my psychological well-being or even checked my stitches… the focus is 100% on the new baby and 0% on mothers.”

The final theme that was identified was related to unsatisfactory postpartum checks for themselves related to their physical recovery. Women mentioned not having any health checks for themselves and if they did, they were short and not much information was provided about their physical healing. Women often commented that they did not receive a physical exam and if they did, they often had to ask and advocate for it. Women who had caesarean sections also commented that they were surprised that they had to wait until 6-weeks to get checked despite having a major surgery. Some mothers also commented that their healthcare provider did not check on their mental health adjustment:

Very focused on the baby and not myself. Given my mental health history, which was even brought up by my OB at our first appointment… I am surprised I was not asked how I was doing… or monitored more closely. I have extreme anxiety and depression at the moment, and now I'm left with no family doctor.

## Discussion

This study found that there is variation in healthcare providers following women during their pregnancy and postpartum, with little consistency in timing and frequency of postnatal visits. Women were found to value in-person follow-up from public health nurses and midwives and were satisfied when they received support from their healthcare providers in a timely, appropriate manner. However, unsatisfactory postpartum care was related to challenges accessing postnatal care, gaps in postnatal follow-ups, and unsatisfactory postpartum checks for themselves related to their own recovery.

The most common time when women had a postnatal follow-up was between day 3–7 for just over half of respondents (57.6%), which was likely a visit from a public health nurse, as included provinces have a public health nurse offer a visit once a woman is home from the hospital. In the Canadian Maternity Survey, most women (93.3%) received a postnatal follow-up visit from a healthcare provider on a mean of 6.9 days (Public Health Agency of Canada, 2009). However, included provinces in this study had longer delays in receiving their first follow-up, with a mean of 7.1 days in PEI, 8.7 days in NS, and 14.6 days in NB (Public Health Agency of Canada, 2009). Additionally, two-thirds of respondents had their first follow-up visit within the first week, with the largest follow-up period between days 3 and 7. Interestingly, approximately 40% of women did not have a follow-up appointment between 6 and 7 weeks, which is an important period for providing information on family planning and providing mental health assessments (Stumbras et al., 2016; Tully et al., 2017). Our results indicate that not much change has happened in almost 14 years, with postnatal follow-ups after birth mostly occurring within the first week and not all women receiving the suggested postpartum follow-up at 6 weeks postpartum. Additionally, primiparous women were more likely to have at least a follow-up appointment in weeks 3, 4, and 6, suggesting their needs may differ than multiparous women, warranting further analysis and exploration.

Women also have challenges accessing timely and adequate postnatal care, due to not having a healthcare provider available (Public Health Agency of Canada, 2009). This also includes challenges related to seeking care for not only themselves but also their newborn. In Canada, 13% of mothers have been found to have had challenges accessing their care provider during a nonroutine appointment (Brandon et al., 2016). A survey in the United States found that there was on average 24.4 min (SD = 11.7) spent per postpartum appointment, with significant variation in the services and assessments provided in the appointments depending on healthcare profession (Krishnamurti et al., 2020). There is significant opportunity for improvements in the care provided to women during the postnatal period.

In our study, three-quarters of women were satisfied with their care, which is lower than the previous Canadian average of 90.9% and the average of 93.5% in these three provinces (Public Health Agency of Canada, 2009). Women who were satisfied with their care reported receiving valuable in-person follow-up from public health nurses and midwives, receiving support from their healthcare providers and receiving timely, appropriate care. Evidence shows that the relationship and care that is provided through midwifery and public health nurses is highly valuable to women and validates their experiences (Aston et al., 2015, 2016; Perriman et al., 2018). As women express satisfaction at the in-person follow-up care provided during the postpartum period, greater consideration of the need to offer at home, in-person follow-up services is warranted. This is especially the case as unsatisfactory postnatal care was related to challenges in accessing postnatal care, gaps in postnatal follow-ups, and having unsatisfactory postpartum checks for their own recovery. In a recent meta-synthesis of over 800 women from 15 countries, what women felt mattered most during the postnatal period was the ability to have a positive motherhood experience, including developing self-esteem, competence, and autonomy, as well as adapting to their new role and relationships while ensuring health for themselves and their infant (Finlayson et al., 2020). In order to achieve these ultimate outcomes, appropriate care and support during the postnatal period is essential.

Studies have shown that during the postnatal period, receiving support and timely, appropriate care is key to a positive experience. In Nova Scotia, Aston et al. (2015) explored in-home visits by public health nurses for new mothers and found that these relationships were essential to new mothers in that the developmental of a good, nonjudgmental relationship is important for this type of support to be successful. Aston et al. (2018) also found that the way that information was shared was important, and that the tone in which the information was provided was imperative. This was also shown in our study as mothers identified challenges with feeling listened to and feeling ignored or rushed during appointments—these behaviors were associated with an unsatisfactory experience of postnatal care. Thus, it is important that postnatal care providers are cognizant of not just the information they are providing but also how they are delivering the care and support.

### Recommendations

More work is needed to ensure that women receive sufficient postnatal care and follow-ups during the immediate 6-week period for both themselves and their newborn. There is a significant paucity of evidence and evaluation on the timing of postnatal visits in Canada and worldwide. As there are no clear postnatal follow-up guidelines, women do not know how often they should be having postpartum care visits for themselves, and healthcare providers are not scheduling follow-up appointments for the mothers the same way well-baby checks are scheduled for newborns. Previous systematic reviews have found inconsistent findings related to improved maternal outcomes for home visits and postpartum support, but there was significant variation in timing and content of these visits (Shaw et al., 2006; Yonemoto et al., 2017). Further research is needed to determine the best timing and frequency of postnatal visits for women and their newborns.

In our study, 40% of women did not report having a postpartum visit in week 6 or 7, which is a concern as the immediate 6 weeks after a birth can be associated with health concerns for the mother and her infant. Additionally, parity influences when women received postnatal care, with primiparous women having more follow-up visits during weeks 3, 4, and 6, suggesting variation in care needs and/or accessibility. Improvements in standardization of the frequency of care for all women, regardless of parity, are recommended to improve the postnatal experience of mothers in these provinces, as well as across Canada.

While the Canadian national postpartum care guidelines were recently released in late 2020 with an increased focus on family-centered care, there was no mention of standardization of postpartum care (Public Health Agency of Canada, 2020). The current guidelines recommend to “plan the timing and purpose of each postpartum contact in partnership with the woman and her partner/family based on their individual needs” and “provide information and support in a timely fashion, according to the needs of the woman, her partner, and family” (Public Health Agency of Canada, 2020, p. 5). While the family-centered care approach is essential, the lack of attention to standardized care delivery leaves room for variation in the provision of postpartum care for women across the country. At a minimum, following WHO (2013) and SOGC (2020) guidelines, women should have a follow-up appointment by 6 weeks to check on mental health adjustment and physical healing from both vaginal and caesarian births. While it is important that postpartum care is women-focused and done in consultation based on the mother's needs and wishes, having a minimum standard of postpartum care is necessary to improve the care that women receive during the postpartum period.

Therefore, our primary recommendations are for (1) guidelines for postpartum visits for women and infants need to be more clearly defined and information disseminated for the Maritime provinces and other areas of Canada and (2) use of these guidelines should receive evaluation in terms of utilization and acceptability to new mothers (and perhaps other family members and health care providers).

### Limitations

While our study helps fill the gap in knowledge related to the postnatal care experience in three provinces in Canada, there are some limitations. First, using a self-report online survey asking women to reflect on their postpartum experience may result in biased or selective reporting. However, by limiting the survey to mothers who had given birth within the past 6 months and the fact that the average age of the infant was 3.2 months helps to ensure that their postpartum experience was relatively recent. We also did not differentiate among respondents in the three provinces which may limit specific recommendations; however, the similarity of postpartum care and representativeness of respondents across the three provinces suggests that our findings should be applicable across all three. The survey was in English and issues may vary when English is not the primary method of communication, as NB is a bilingual province. Finally, we did not ask how many times respondents saw each type of healthcare provider nor did we ask whether the visit was for the mother or the newborn. This could have shed some additional light on the number of and types of postnatal interactions.

Our work builds on the Maternity Experience Survey findings (Chalmers et al., 2008; Public Health Agency of Canada, 2009) to provide current and further insight into the postnatal experiences of Canadian women. Given that there is currently no standardization of care across Canada, similar postnatal experiences may exist and there is possibility of generalization to the Canadian population. However, this should be done in consideration of the potential differences in postnatal care provided in each province. Further study is warranted to determine whether the findings are similar across Canada as well as other countries.

## Conclusion

There is variation across the three Eastern Canadian provinces in relation to provision of follow-up care during the postnatal period—in terms of healthcare providers as well as the timing and frequency of postnatal visits. While many women are experiencing satisfactory care, there are areas where women are reporting dissatisfaction and are facing challenges. Improvements are needed to the postpartum care experience of mothers, including but not limited to, standardization of postnatal care contacts and the need for provision of personalized care that addresses women's health needs and provides support, and enhancing timely, appropriate care for herself and the newborn. The findings highlight the variability that exists within provincial regions as well as across provinces, highlighting the need for the national and provincial consensus guiding postnatal care for the women and her newborn.

## References

[bibr1-08445621211052141] Aston M. Etowa J. Price S. Vukic A. Hart C. MacLeod E. Randel P. (2016). Public health nurses and mothers challenge and shift the meaning of health outcomes. Global Qualitative Nursing Research, 3, 1–10. 10.1177/2333393616632126 PMC5342652 28462331

[bibr2-08445621211052141] Aston M. Price S. Etowa J. Vukic A. Young L. Hart C. MacLeod E. Randel P. (2015). The power of relationships: Exploring How public health nurses support mothers and families during postpartum home visits. Journal of Family Nursing, 21(1), 11–34. 10.1177/1074840714561524 25492494

[bibr3-08445621211052141] Aston M. Price S. Monaghan J. Sim M. Hunter A. Little V. (2018). Navigating and negotiating information and support: Experiences of first-time mothers. Journal of Clinical Nursing, 27(3–4), 640–649. 10.1111/jocn.13970 28722771

[bibr4-08445621211052141] Bradshaw C. Atkinson S. Doody O. (2017). Employing a qualitative description approach in health care research. Global Qualitative Nursing Research, 4, 1–8 10.1177/2333393617742282 PMC5703087 29204457

[bibr5-08445621211052141] Brandon A. D. Costanian C. Sayed M. F. E. Tamim H. (2016). Factors associated with difficulty accessing health care for infants in Canada: Mothers’ reports from the cross-sectional maternity experiences survey. BMC Pediatrics, 16(192), 1–9. 10.1186/s12887-016-0733-4 27887580 PMC5124240

[bibr6-08445621211052141] Braun V. Clarke V. (2006). Using thematic analysis in psychology. Qualitative Research in Psychology, 3(2), 77–101. https://doi.org/dx.https://doi.org/10.1191/1478088706qp063oa

[bibr7-08445621211052141] Canadian Paediatric Society (2020). *Schedule of well-child visits*. Webpage. Accessed October 1, 2021 from https://www.caringforkids.cps.ca/handouts/schedule_of_well_child_visits.

[bibr8-08445621211052141] Chalmers B. Dzakpasu S. Heaman M. Kaczorowski J. (2008). The Canadian maternity experiences survey: An overview of findings. Journal of Obstetrics and Gynaecology Canada, 30(3), 217–228. 10.1016/S1701-2163(16)32758-X 18364099

[bibr9-08445621211052141] Daoud N. O’Brien K. O’Campo P. Harney S. Harney E. Bebee K. Bourgeois C. Smylie J. (2019). Postpartum depression prevalence and risk factors among indigenous, non-indigenous and immigrant women in Canada. Canadian Journal of Public Health, 110(4), 440–452. 10.17269/s41997-019-00182-8 30767191 PMC6964473

[bibr10-08445621211052141] Dennis C. L. Falah-Hassani K. Brown H. K. Vigod S. N. (2016). Identifying women at risk for postpartum anxiety: A prospective population-based study. Acta Psychiatrica Scandinavica, 134(6), 485–493. 10.1111/acps.12648 27639034

[bibr11-08445621211052141] Falah-Hassani K. Shiri R. Vigod S. Dennis C. L. (2015). Prevalence of postpartum depression among immigrant women: A systematic review and meta-analysis. Journal of Psychiatric Research, 70, 67–82. 10.1016/j.jpsychires.2015.08.010 26424425

[bibr12-08445621211052141] Finlayson K. Crossland N. Bonet M. Downe S. (2020). What matters to women in the postnatal period: A meta-synthesis of qualitative studies. PLoS ONE, 15(4), 1–23. 10.1371/journal.pone.0231415 PMC7176084 32320424

[bibr13-08445621211052141] Government of Canada (2020). *Health care in Canada*. Webpage. Accessed October 1, 2021 from https://www.canada.ca/en/immigration-refugees-citizenship/services/new-immigrants/new-life-canada/health-care-card.html.

[bibr14-08445621211052141] Haque N. Khanlou N. Skinner A. (2017). Scoping review on maternal health among immigrants and visible minority women in Canada: Postnatal care. Journal of Pregnancy, 2017, 1–14. 10.1155/2017/8783294 PMC5292182 28210508

[bibr15-08445621211052141] Kingston D. Kehler H. Austin M. P. Mughal M. K. Wajid A. Vermeyden L. Benzies K. Brown S. Stuart S. Giallo R. (2018). Trajectories of maternal depressive symptoms during pregnancy and the first 12 months postpartum and child externalizing and internalizing behavior at three years. PLoS ONE, 13(4), 1–19. 10.1371/journal.pone.0195365 PMC5898728 29652937

[bibr16-08445621211052141] Krishnamurti T. Simhan H. N. Borrero S. (2020). Competing demands in postpartum care: A national survey of U.S. providers’ priorities and practice. BMC Health Services Research, 20(1), 1–10. 10.1186/s12913-020-05144-2 PMC7137294 32252757

[bibr17-08445621211052141] Lemyre B. Jefferies A. L. O’Flaherty P. (2018). Facilitating discharge from hospital of the healthy term infant. Paediatrics and Child Health, 23(8), 515–531. 10.1093/pch/pxy127 PMC6242107 30894791

[bibr18-08445621211052141] Norhayati M. N. Nik Hazlina N. H. Asrenee A. R. Wan Emilin W. M. A. (2015). Magnitude and risk factors for postpartum symptoms: A literature review. Journal of Affective Disorders, 175, 34–52. 10.1016/j.jad.2014.12.041 25590764

[bibr19-08445621211052141] Perriman N. Davis D. L. Ferguson S. (2018). What women value in the midwifery continuity of care model: A systematic review with meta-synthesis. Midwifery, 62(April), 220–229. 10.1016/j.midw.2018.04.011 29723790

[bibr20-08445621211052141] Public Health Agency of Canada (2009). What Mothers Say: The Canadian Maternity Experiences Survey. Accessed October 1, 2021 from http://www.publichealth.gc.ca/mes.

[bibr21-08445621211052141] Public Health Agency of Canada (2020). Chapter 5: Postpartum care. In Family-centred maternity and newborn care: National guidelines. PHAC. 1–85. https://www.canada.ca/content/dam/hc-sc/documents/services/publications/healthy-living/maternity-newborn-care-guidelines-chapter-5/maternity-guidelines-chapter-5-en.pdf

[bibr22-08445621211052141] Reproductive Care Program of Nova Scotia (2002). *Healthy Babies, Healthy Families: Postpartum & Postnatal Guidelines*. 1–116. Accessed October 1, 2021 from http://novascotia.ca/dhw/publications/Public-Health-Education/Postpartum Guidelines.pdf.

[bibr23-08445621211052141] Sample Size Calculator (2014). Raosoft, Inc. Accessed October 1, from http://www.raosoft.com/samplesize.html.

[bibr24-08445621211052141] Shaw E. Levitt C. Wong S. Kaczorowski J. (2006). Systematic review of the literature on postpartum care: Effectiveness of postpartum support to improve maternal parenting, mental health, quality of life, and physical health. Birth, 33(3), 210–220. 10.1111/j.1523-536X.2006.00106.x 16948721

[bibr25-08445621211052141] Shorey S. Chee C. Y. I. Ng E. D. Chan Y. H. Tam W. W. S. Chong Y. S. (2018). Prevalence and incidence of postpartum depression among healthy mothers: A systematic review and meta-analysis. Journal of Psychiatric Research, 104(July), 235–248. 10.1016/j.jpsychires.2018.08.001 30114665

[bibr26-08445621211052141] Society of Obstetricians and Gynaecologists of Canada (2020). *Postpartum health care and 6-week postpartum visit*. Webpage. https://www.pregnancyinfo.ca/postpartum/postpartum/postpartum-health-care-and-6-week-postpartum-visit/. Accessed October 1, 2021.

[bibr27-08445621211052141] Statistics Canada (n.d.). *Live births, by place of residence of mother*. 2020. Retrieved June 10, 2020, from https://www150.statcan.gc.ca/t1/tbl1/en/tv.action?pid = 1310041401&pickMembers%5B0%5D = 2.8.

[bibr28-08445621211052141] Statistics Canada (2019). *Annual demographic estimates: Canada, provinces and territories*. Retrieved October 1, 2021 from https://www150.statcan.gc.ca/n1/pub/91-215-x/91-215-x2019001-eng.htm.

[bibr29-08445621211052141] Stumbras K. Rankin K. Caskey R. Haider S. Handler A. (2016). Guidelines and interventions related to the postpartum visit for Low-risk postpartum women in high and upper middle income countries. Maternal and Child Health Journal, 20(1), 103–116. 10.1007/s10995-016-2053-6 27392705

[bibr30-08445621211052141] Sword W. Watt S. Krueger P. Thabane L. Landy C. K. Farine D. Swinton M. (2009). The ontario mother and infant study (TOMIS) III: A multi-site cohort study of the impact of delivery method on health, service use, and costs of care in the first postpartum year. BMC Pregnancy and Childbirth, 9(16), 1–12. 10.1186/1471-2393-9-16 19397827 PMC2688481

[bibr31-08445621211052141] Tully K. P. Stuebe A. M. Verbiest S. B. Parides M. Bickell N. Leventhal H. (2017). The fourth trimester: A critical transition period with unmet maternal health needs. American Journal of Obstetrics and Gynecology, 127(0), e86–e92. 10.1016/j.ajog.2017.03.032 28390671

[bibr32-08445621211052141] Verbiest S. Bonzon E. Handler A. (2016). Postpartum health and wellness: A call for quality woman-centered care. Maternal and Child Health Journal, 20(1), 1–7. 10.1007/s10995-016-2188-5 27757754

[bibr33-08445621211052141] World Health Organization (2010). *WHO technical consultation on postpartum and postnatal care*. Accessed October 1, 2021 from http://apps.who.int/iris/bitstream/handle/10665/70432/WHO_MPS_10.03_eng.pdf?sequence = 1%0A http://www.who.int/maternal_child_adolescent/documents/WHO_MPS_10_03/en/

[bibr34-08445621211052141] World Health Organization (2013). Postnatal care of the mother and newborn 2013. World Health Organization. Accessed October 1, 2021 from https://doi.org/9789241506649 24624481

[bibr35-08445621211052141] Yonemoto N. Dowswell T. Nagai S. Mori R. (2017). Schedules for home visits in the early postpartum period. Cochrane Library, 7, 1–71. 10.1002/14651858.CD009326.pub2 PMC6483560 28770973

